# ESBL-producing *Atlantibacter hermannii* harboring a plasmid encoding IS*26* up and down stream of *bla*_CTX-M-27_ ,*bla*_LAP-2_, and *qnrS1* isolated from an edible river *Anabas testudineus* fish

**DOI:** 10.1128/mra.00056-24

**Published:** 2024-04-30

**Authors:** Michio Jinnai, Takahiro Yamaguchi, Doan Tran Nguyen Minh, Oanh Nguyen Hoang, Hien Le Thi, Phong Ngo Thanh, Phuong Hoang Hoai, Phuc Nguyen Do, Chinh Dang Van, Yuko Kumeda, Astushi Hase, Tatsuya Nakayama

**Affiliations:** 1 Department of Microbiology, Kanagawa Prefectural Institute of Public Health, Chigasaki, Kanagawa, Japan; 2 Division of Microbiology, Osaka Institute of Public Health, Higashinari, Osaka, Japan; 3 Institute of Public Health Ho Chi Minh City, Ho Chi Minh City, Vietnam; 4 Research Center of Microorganism Control, Osaka Metropolitan University, Sakai, Osaka, Japan; 5 Faculty of Contemporary Human Life Science, Tezukayama University, Nara, Japan; 6 Graduate School of Integrated Sciences for Life,, Hiroshima University, Higashi-Hiroshima, Hiroshima, Japan; University of Maryland School of Medicine, Baltimore, Maryland, USA

**Keywords:** *Atlantibacter hermannii*, *bla*_CTX-M-27_, *qnrS1*, IS*26*

## Abstract

Extended-spectrum β-lactamase-producing *Atlantibacter hermannii* was isolated from an edible river fish, *Anabas testudineus*, which was sold in a market located in Vietnam. The genome sequence was obtained by using next-generation sequencing, which involved Oxford Nanopore and Illumina technologies. The 92 kb plasmid encodes the gene *bla*_CTX-M-27_.

## ANNOUNCEMENT

Extended-spectrum β-lactamases (ESBL)-producing bacteria have been detected in various food products, making food an important source of human infection (1). To date, few reports have been made on the complete genome of *Atlantibacter hermannii* encoding the ESBL-related gene. Here, we report the whole genome sequence of ESBL-producing *A. hermannii* isolated from purchased edible *Anabas testudineus* fish from retail stores in Ho Chi Minh City.

The isolation source, the fish, which was purchased between 10 March 2020 and 19 March 2020, was processed on the same day of purchase and had not been frozen. Five gram of fish gut contents was mixed with 45 mL of buffered peptone water and incubated at 37°C for 22 h. Ten microliter of the bacterial broth was inoculated on CHROMagar ECC (CHROMagar, Paris, France) with 2 mg/L cefotaxime. The mauve colonies were picked up and confirmed for ESBL production (2). The selected bacterium was identified as *A. hermannii* using matrix-assisted laser desorption/ionization-time of flight mass spectrometry (Bruker, Bremen, Germany). DNA was extracted from the bacterium grown on Mueller-Hinton agar using NucleoSpin Microbial DNA (Takara, Shiga, Japan) for short-read and NucleoBond AXG column with Buffer Set III (Takara) for long-read sequencing according to the manufacturer’s instructions. A Qubit dsDNA High Sensitivity Assay Kit (Thermo Fisher, Waltham, USA) was used to analyze the extracted DNA. The QIAseq FX DNA library UDI kit (Qiagen, Hilden, Germany) and Rapid Barcoding Kit (Oxford Nanopore, Oxford, UK) were used for short- and long-read sequencing library preparation, respectively. Short- and long-read sequencing were carried out using the Illumina iSeq 100 (Illumina, San Diego, USA) with a 2 × 150 bp paired-end protocol and MinION with flow cell R9.4.1 (Oxford Nanopore), respectively. Guppy v5.0.11 was used as the base caller of long-read sequencing with super-accuracy base calling. The short- and long-read sequences were trimmed using fastp v0.23.2and NanoFilt v2.8.0, respectively (3). Quality checks were performed on the short- and long-read sequences using fastQC v0.12.1and Nanoplot v1.41.0 (3), respectively. The trimmed long-read sequence (total reads 79,686; read length N50 13,707 bp; total bases 655.1 Mb; coverage 139.5×) was assembled and circularized by flye v2.9.2 and polished with the trimmed short-read sequence [total reads 31,831,940; mean length after filtering 2 × 145 bp reads (pair end); total bases 462.2 Mb; coverage 98.2×] by Pilon v1.24, BWA v0.7.17, and samtools v1.6 (4). Default parameters were used for all software. The genome sequence was assembled into four circular contigs (Table 1), identified as *A. hermannii* by DFAST (dfast.ddbj.nig.ac.jp), and analyzed using ResFinder v4.4.2 and Mobile Element Finder v1.1.2. All four contigs were rotated by DFAST.

**TABLE 1:** Genome information of *A. hermannii* VNF293

	Chromosome	Plasmid 1	Plasmid 2	Plasmid 3	Total
Strain name	VNF293	pVNF293-1	pVNF293-2	pVNF293-3	
Length (bp)	4,564,762	92,069	4,892	34,490	4,696,213
GC content (%)	54.0	52.5	45.2	52.4	54.0
No. of CDS	4,205	103	4	56	4,368
No. of rRNA	22	0	0	0	22
No. of tRNA	84	0	0	0	84
Mobile genetic element	IS*30*-like and IS*Spr2*-like	IS*6100*, IS*26*-like, IS*26*, and IS*As17*-like			
Antibiotic resistance gene	*bla*_HERA-8_-like and *catA1*	*bla*_CTX-M-27_, *bla*_LAP-2_, *qnrS1*, and *dfrA14*			

**Fig 1:**
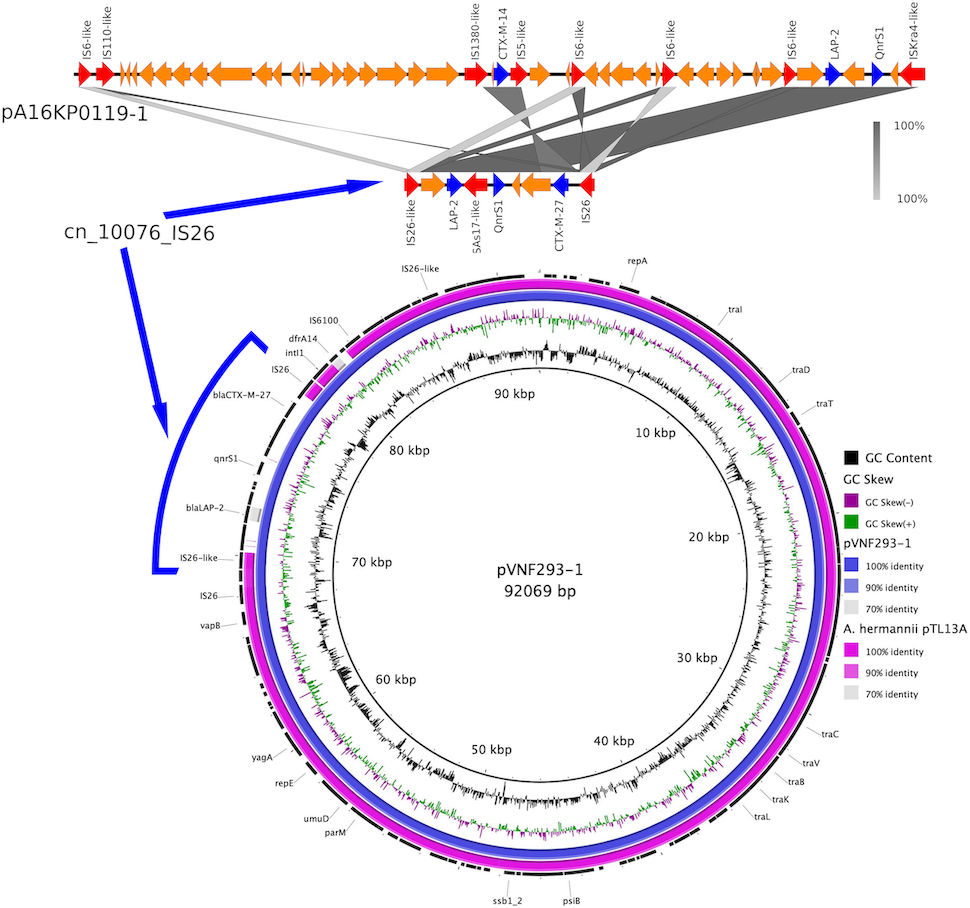
The genetic structure of pVNF293-1 harbored by *A. hermannii*. The complete genome of *A. hermannii* p13TLA (accession number CP126340.1) and *K. pneumoniae* p16AKp0119-1 (accession number CP052570.1) was selected for the plasmid map of pVNF293-1 and comparison with a putative composite transposon (cn_10076_IS26), respectively. BLAST Ring Image Generator v0.95 was used to design plasmid maps. In the image comparison of cn_10076_IS26 and pA16KP0119-1, red arrows represent mobile elements, and blue arrows represent antibiotic-resistance genes.

The plasmid pVNF293-1 with 93,609 bp has 99.99% identity to plasmid p13TLA of *A. hermannii*, which was previously reported. The pVNF293-1 carries a putative transposon cn_10076_IS26 with *bla*_LAP-2_, *bla*_CTX-M-27_, and *qnrS1* genes flanked by IS*26*-like elements and IS*26* (Table 1; Fig. 1). The cn_10076_IS26 showed 99.94% identity with a plasmid, pA16KP0119-1, harbored by *Klebsiella pneumoniae* (accession number CP052570.1). These results may indicate the horizontal transmission of *bla*_CTX-M-27_ between *A. hermannii* and other Enterobacterales.

## Data Availability

The complete sequences of A. hermannii were deposited in GenBank (accession numbers AP028957–AP028960). The raw reads were deposited under the accession numbers DRR505221 and DRR505222 in BioProject number PRJDB16699.
